# Evaluation of the Effectiveness of Student Practice in a Surgical Skills Curriculum: A Prospective Study

**DOI:** 10.7759/cureus.81996

**Published:** 2025-04-10

**Authors:** Diogo S Almeida, Silvio P Junior, Gileno Falcon, José Humberto O Campos, Victor A Felzemburgh, Leonardo B Dourado, Luis Henrique Medina, Malik P Prates, Henrique A Matos, Mary Silva

**Affiliations:** 1 General Surgery, Bahiana School of Medicine and Public Health, Salvador, BRA; 2 Plastic Surgery, Bahiana School of Medicine and Public Health, Salvador, BRA; 3 General Surgery, Zarns Health Education Institute, Salvador, BRA; 4 Emergency Medicine, Bahiana School of Medicine and Public Health, Salvador, BRA; 5 Medicine, Bahiana School of Medicine and Public Health (EBMSP), Salvador, BRA

**Keywords:** knowledge retention, medical education, procedural training, student practice, surgical skills

## Abstract

Objectives: This study aims to evaluate the effectiveness of student practice in a surgical skills curriculum and their evolution during the process and, therefore, evaluate the relevance of training surgical skills in medical graduation.

Methods: The study design was prospective observational and analytical, the participants were fourth-year medical students from the Bahiana School of Medical Education and Public Health, and the sample size was 300. The sampling method used was a non-probability sampling method, and data collection was realized through a questionnaire in a QR code with a study overview and a pre-assessment and post-assessment survey link. Data analysis was performed using Microsoft Excel 2021 (Microsoft Corp., Redmond, WA, US). The Shapiro-Wilk test was first conducted to test for normality. Due to the normal distribution of the data, a parametric paired t-test with a 95% confidence interval was used to analyze statistical differences in diagnostic accuracy and knowledge acquisition scores between the pre- and post-assessment and provided the collection of data to assess the impact of a second review moment of surgical skills (SRMSS) for medical students.

Results: Perceived readiness to teach improved across all weeks. Pre- and post-assessment scores showed significant increases (p < 0.05), with mean differences ranging from 0.89 to 1.32, the largest change occurring in Week 1 (1.32). Perceived readiness to apply topics in professional life improved across all weeks, with mean differences ranging from 0.52 to 1.32 (p < 0.05).

Conclusion: The results show that the SRMSS implemented over the six-week period were effective in enhancing participants' perceived learning, readiness for assessments, readiness to teach, and readiness to apply learned topics in practical or professional contexts.

## Introduction

Surgical skills are a fundamental component of medicine in Brazil, since non-specialized doctors end up needing to perform minor surgical procedures in their routine care, playing a critical role in the treatment of a variety of medical conditions. The ability to perform surgical procedures with dexterity and precision not only ensures the success of treatment but also directly influences patient recovery and quality of life. However, acquiring these skills requires carefully structured practical training. This is where the importance of a dedicated curricular component for such training becomes evident. Through this component, medical students have the opportunity to learn and practice surgical techniques in a supervised environment, not only acquiring technical skills but also developing the confidence and ethics necessary for responsible surgical practice; therefore, the objective of this study is to evaluate the evolution of the students’ habilities and confidence, after supervised training of surgical skills.

This practical training not only shapes competent surgeons but also ensures patient safety. Early exposure to real surgical situations allows students to make mistakes and learn from them, under the guidance of experienced professionals. This results in more qualified and aware healthcare providers, capable of offering high-quality surgical care. Moreover, by providing a solid foundation in surgical skills, this curricular component prepares future physicians to face the challenges and complexities of modern medicine, thus contributing to excellence in the field of medical surgery.

The relevance of practice in the educational journey of medical students, particularly in the context of surgical skills, is a prominent theme in medical and educational literature. The training of surgeons requires a holistic approach that encompasses robust theoretical knowledge while prioritizing practical experience, given the highly technical and delicate nature of surgery.

The literature emphasizes that the acquisition of surgical skills cannot be achieved solely through theoretical lectures or simulator training [1,2]. While simulators provide valuable initial exposure [3], real clinical environments are crucial for developing the dexterity, coordination, and precision required to perform surgical procedures safely and effectively. These hands-on experiences bridge the gap between theoretical knowledge and real-world application.

Studies indicate that medical students who actively participate in surgeries during their training perform significantly better in surgical procedures compared to those who lack such experience [4]. Surgical practice allows students to gain real-time familiarity with anatomy, understand technical challenges, and learn how to manage unforeseen complications during surgeries. Moreover, direct participation in surgeries reinforces their ability to integrate theoretical knowledge with procedural execution, fostering confidence and competence and avoiding the occurrence of complications due to inexperience and incompetence, which are the most common causes of errors in some teaching hospitals [5].

Beyond technical proficiency, surgical practice fosters the development of non-technical skills such as teamwork, effective communication, and decision-making under pressure [6,7]. These skills are critical for surgeons, as surgery often involves collaboration with other healthcare professionals and necessitates quick, accurate decision-making during complex procedures. For instance, navigating intraoperative complications requires both individual skill and coordinated teamwork to ensure patient safety and optimal outcomes.

Early exposure to surgical practice helps students determine whether surgery is a field they wish to specialize in, enabling them to make informed career choices [8,9]. Such experiences provide valuable insights into the demands and rewards of surgical careers, aiding in self-assessment and career planning. Additionally, surgical practice offers opportunities to internalize surgical ethics, such as informed consent, respect for patient autonomy, and adherence to the principles of beneficence and non-maleficence. Ethical training ensures that students not only master surgical techniques but also uphold the highest standards of patient care.

It is essential that surgical practice be supervised by experienced surgeons and conducted in compliance with ethical and safety standards. The literature underscores the necessity of a structured and safe learning environment to ensure students gain maximum benefits from surgical practice while minimizing risks to patients [10,11]. Supervision by skilled mentors not only safeguards patient welfare but also facilitates constructive feedback and skill refinement.

Through hands-on experiences, students not only refine their skills but also cultivate the confidence and decision-making capabilities essential for surgical excellence. By fostering ethical awareness and encouraging collaborative teamwork, surgical training ensures that students are well-equipped to navigate the complexities of patient care. As institutions continue to evolve their training programs, a steadfast commitment to structured, supervised, and ethically grounded surgical practice will remain vital in preparing the next generation of competent and compassionate surgeons.

## Materials and methods

Study design, sample size, and participants

After the Research Ethics Committee of the Bahiana School of Medical Education and Public Health provided ethical approval, following the Helsinki Declaration guidelines, a prospective assessment was performed to assess the impact of a second review moment of surgical skills (SRMSS) for medical students from the Bahiana School of Medical Education and Public Health. The study took place in a training lab of surgical skills. To ensure the privacy and safety of the participants, ethical considerations were taken into account in this study. Informed consent was obtained from all participants prior to their involvement in the study, with a clear explanation of the research proposition and procedures. Confidentiality was maintained throughout the study by anonymizing participant data.

All fourth-year medical students enrolled during June of 2023 and July of 2024 (n = 300) were invited to participate in the study. All students received a QR code with a study overview and a pre-assessment and post-assessment survey link. The pre-assessment and post-assessment survey included four questions, and the students had to classify them from 0 to 10: (1) How much do you feel you have learned about this week's topics? (2) How prepared do you believe you are to conduct a practical assessment on these topics? (3) How prepared do you believe you are to teach these topics to a colleague? (4) How prepared do you believe you are to apply the knowledge about these topics in your practical/professional life? Students who did not answer both pre- and post-assessment were excluded.

Of the 300 surveyed students, 119 (response rate = 39.6%) completed the survey. The participants were a diverse group of fourth-year medical students with various educational backgrounds, consisting of 68 women and 51 men, all interested in pursuing a medical degree. The study was voluntary, and participants were not compensated.

Weeks' themes

The surgical themes during the weeks were the following: Week 1: Surgical Environment, Hand Washing, Gowning, Gloving, Patient Preparation, Surgical Drapes, and Team Positioning; Week 2: Instrument Table Setup, Signaling, Handling of Materials, Fundamental Maneuvers, and Special Materials; Week 3: Surgical Needles, Surgical Sutures, Knots, Clamping, and Ligatures; Week 4: Sutures, Wound Cleaning, and Local Anesthesia; Week 5: Catheters and Drains; and Week 6: Injections, Peripheral Access, Intraosseous Puncture, Arterial Puncture, and Central Venous Access.

Intervention, data collection, and outcome measures

In the SRMSS, there were a total of 60 students for each week (a sample size of 300 students), where they could practice the themes (which they had theoretical-practical lessons previously) of the weeks for a total of one hour and 30 minutes, using synthetic prototypes. These participants were asked to answer two identical questionnaires: the first before the SRMSS and the second after the SRMSS. These questionnaires assessed the students' subjective learning about these surgical skills.

Analysis

All statistical tests were performed using Microsoft Excel 2021 (Microsoft Corp., Redmond, WA, US). The Shapiro-Wilk test was first conducted to test for normality. Due to the normal distribution of the data, a parametric paired t-test with a 95% confidence interval was used to analyze statistical differences in diagnostic accuracy and knowledge acquisition scores between the pre- and post-assessment.

## Results

Characteristics of the students

A total of 119 students answered the pre- and post-assessment over the weeks. There were a total of 51 men (42.8%) and 68 women (57.2%).

Perceived learning 

As presented in Table 1, all weekly sessions showed a statistically significant improvement in perceived learning (p < 0.05). The lowest mean difference was observed in Week 2 (mean difference = 0.55), while the highest gain occurred in Week 6 (mean difference = 1.09). This progressive increase suggests that the SRMSS enhanced students' confidence in their understanding of the topics covered. Notably, Weeks 5 and 6 showed the greatest post-intervention increases, possibly reflecting the cumulative effect of repeated practice and familiarity with procedural content.

**Table 1:** Perceived Learning

	Mean pre-assessment	Mean post-assessment	p-value
Week 1	7.79 (±1.30)	8.53 (±1.06)	<0.05
Week 2	7.84 (±1.49)	8.39 (±1.36)	<0.05
Week 3	7.76 (±1.31)	8.47 (±1.14)	<0.05
Week 4	8.01 (±1.32)	8.81 (±1.12)	<0.05
Week 5	7.25 (±1.37)	8.29 (±1.33)	<0.05
Week 6	7.21 (±1.56)	8.30 (±1.19)	<0.05

Perceived readiness for conducting a practical assessment

Table 2 highlights statistically significant increases in students’ perceived readiness for conducting assessments after the SRMSS sessions (p < 0.05 for all weeks). The smallest gain was in Week 2 (mean difference = 0.63), and the largest was in Week 6 (mean difference = 1.11). These findings reflect improved self-confidence in performing surgical tasks and indicate growing readiness for practical evaluation.

**Table 2:** Perceived Readiness for Conducting a Practical Assessment

	Mean pre-assessment	Mean post-assessment	p-value
Week 1	6.85 (±1.95)	7.95 (±1.47)	<0.05
Week 2	7.52 (±1.68)	8.15 (±1.42)	<0.05
Week 3	6.82 (±1.98)	7.88 (±1.54)	<0.05
Week 4	7.79 (±1.47)	8.70 (±1.21)	<0.05
Week 5	7.14 (±1.44)	8.13 (±1.40)	<0.05
Week 6	7.05 (±1.70)	8.16 (±1.29)	<0.05

Perceived readiness to teach these topics

Table 3 demonstrates that students felt significantly more prepared to teach surgical topics following each session. Weeks 1 and 3 both recorded the largest increase in perceived teaching readiness (mean difference = 1.32), possibly due to the foundational nature of the early sessions and their clarity. All results were statistically significant (p < 0.05).

**Table 3:** Perceived Readiness to Teach These Topics

	Mean pre-assessment	Mean post-assessment	p-value
Week 1	6.59 (±2.36)	7.91 (±1.64)	<0.05
Week 2	7.18 (±1.84)	8.07 (±1.49)	<0.05
Week 3	6.52 (±2.41)	7.84 (±1.74)	<0.05
Week 4	7.67 (±1.51)	8.65 (±1.25)	<0.05
Week 5	7.0 (±1.52)	8.13 (±1.42)	<0.05
Week 6	7.03 (±1.69)	8.11 (±1.32)	<0.05

Perceived readiness to apply these topics in the practical/professional life

In Table 4, all weeks again show statistically significant improvements (p < 0.05). The largest difference was in Week 6 (mean difference = 1.15), suggesting that recent exposure and practice may have had the strongest effect on students' perceived real-world readiness. The smallest difference was in Week 2 (mean difference = 0.52), once again indicating potential limitations in engagement or applicability of those earlier cognitive-heavy topics.

**Table 4:** Perceived Readiness to Apply These Topics in the Practical/Professional Life

	Mean pre-assessment	Mean post-assessment	p-value
Week 1	6.59 (±2.36)	7.91 (±1.64)	<0.05
Week 2	7.42 (±1.76)	7.94 (±1.45)	<0.05
Week 3	7.0 (±2.17)	7.95 (±1.65)	<0.05
Week 4	7.73 (±1.65)	8.51 (±1.39)	<0.05
Week 5	6.94 (±1.67)	7.98 (±1.70)	<0.05

## Discussion

The objective of this study was to assess the impact of SRMSS on medical students. Four dimensions were analyzed: (1) perceived learning, (2) perceived readiness for conducting a practical assessment, (3) perceived readiness to teach these topics, and (4) perceived readiness to apply these topics in professional practice.

All aspects showed a statistically significant increase in post-intervention evaluation scores (p < 0.05), suggesting that SRMSS contributed positively to self-perceived gains in surgical skill domains. Weekly average scores also increased across all dimensions, reinforcing the potential benefits of structured, repeated practice sessions, as previously suggested by similar studies [12].

Notably, the smallest increases were observed in Week 2 (Instrument Table Setup, Signaling, Handling of Materials, Fundamental Maneuvers, and Special Materials), which may reflect the more cognitive and memorization-based nature of these topics. In contrast, higher gains were noted in Weeks 4 and 5, particularly for suturing and procedural techniques, likely due to their more hands-on, motor-skill-intensive nature.

The practice of these medical skills may also be related to the success rate of the procedures tested. An example of this can be seen in the study by İskender and Karadeniz [13], which conducted a systematic review with meta-analysis evaluating the difference between a group that underwent training versus a group that did not train for central venous cannulation. This study showed that the trained group had a better development.

Supporting this, Hanada et al. [14] and Denadai et al. [15] have demonstrated that supervised practice significantly enhances performance in suturing, both in time efficiency and technical quality. Moreover, Offiah et al. [16] emphasized that continuous practice is essential for long-term retention and clinical application of technical skills, such as venous cannulation and catheterization.

Despite these promising findings, this study has important limitations. First, all measurements were based on self-perceived outcomes, rather than objective assessments. This introduces a subjectivity bias, as participants may overestimate or underestimate their competence. Second, the absence of a control group limits causal inferences. Third, the sample was drawn from a single institution, which may restrict the generalizability of results.

## Conclusions

The implementation of SRMSS over a six-week period was associated with improvements in participants' perceived learning, readiness for assessments, teaching ability, and applicability in real-world clinical settings. However, as the results are based on subjective self-assessment, further studies employing objective performance metrics are essential to confirm the effectiveness of such interventions. Additionally, future research should explore the long-term impact of review sessions and assess whether such improvements translate into real clinical proficiency and patient safety outcomes.

## Appendices

Pre-assessment questionnaire

1. Approximately how much time did you spend studying these topics previously? (Including video classes, workshops, prior study, and training)

- Less than 1 hour

- 1-2 hours

- 2-4 hours

- 4-6 hours

- More than 6 hours

2. How much do you identify that you learned about the topics before practice time? (Scale: 0 = no learning, 10 = complete learning)

- Number answer

3. How prepared do you believe you are to carry out a practical assessment on these topics before practice time? (Scale: 0 = unprepared, 10 = completely prepared)

- Number answer

4. How prepared do you think you are to teach these topics to a colleague before practice time? (Scale: 0 = unprepared, 10 = completely prepared)

- Number answer

5. How prepared do you believe you are to apply the knowledge on this topic in your practical/professional life before practice time? (Scale: 0 = unprepared, 10 = completely prepared)

- Number answer

Post-assessment questionnaire

1. How much do you identify that you learned about the topics after practice time? (Scale: 0 = no learning, 10 = complete learning)

- Number answer

2. How prepared do you believe you are to carry out a practical assessment on these topics after practice time? (Scale: 0 = unprepared, 10 = completely prepared)

- Number answer

3. How prepared do you think you are to teach these topics to a colleague after practice time? (Scale: 0 = unprepared, 10 = completely prepared)

- Number answer

4. How prepared do you believe you are to apply the knowledge on this topic in your practical/professional life after practice time? (Scale: 0 = unprepared, 10 = completely prepared)

- Number answer

## References

[1] Ericsson KA, Krampe RT, Tesch-Römer C (1993). The role of deliberate practice in the acquisition of expert performance. Psychol Rev.

[2] Kneebone R (2010). Simulation, safety and surgery. Qual Saf Health Care.

[3] Tsuda S, Scott D, Doyle J, Jones DB (2009). Surgical skills training and simulation. Curr Probl Surg.

[4] Stefanidis D, Acker C, Heniford BT (2008). Proficiency-based laparoscopic simulator training leads to improved operating room skill that is resistant to decay. Surg Innov.

[5] Gawande AA, Zinner MJ, Studdert DM, Brennan TA (2003). Analysis of errors reported by surgeons at three teaching hospitals. Surgery.

[6] Fried MP, Satava R, Weghorst S (2004). Identifying and reducing errors with surgical simulation. Qual Saf Health Care.

[7] Hull L, Arora S, Kassab E, Kneebone R, Sevdalis N (2011). Assessment of stress and teamwork in the operating room: an exploratory study. Am J Surg.

[8] Antiel RM, Reed DA, Van Arendonk KJ (2013). Effects of duty hour restrictions on core competencies, education, quality of life, and burnout among general surgery interns. JAMA Surg.

[9] Zuccato JA, Kulkarni AV (2016). The impact of early medical school surgical exposure on interest in neurosurgery. Can J Neurol Sci.

[10] Dedy NJ, Szasz P, Louridas M, Bonrath EM, Husslein H, Grantcharov TP (2015). Objective structured assessment of nontechnical skills: reliability of a global rating scale for the in-training assessment in the operating room. Surgery.

[11] Reznick RK, MacRae H (2006). Teaching surgical skills--changes in the wind. N Engl J Med.

[12] Alanazi AA, Nicholson N, Thomas S (2017). The use of simulation training to improve knowledge, skills, and confidence among healthcare students: a systematic review. Internet J Allied Health Sci Pract.

[13] İskender N, Karadeniz H (2025). The effect of central venous catheter care training following evidence-based guidelines on nurses' knowledge levels and care practices. J Eval Clin Pract.

[14] Hanada K, Hoshina K, Tsuyuki S (2022). Ten-hour simulation training improved the suturing performance of medical students. Ann Vasc Surg.

[15] Denadai R, Toledo AP, Oshiiwa M, Saad-Hossne R (2013). Acquisition of suture skills during medical graduation by instructor-directed training: a randomized controlled study comparing senior medical students and faculty surgeons. Updates Surg.

[16] Offiah G, Ekpotu LP, Murphy S (2019). Evaluation of medical student retention of clinical skills following simulation training. BMC Med Educ.

